# Degree of Hydrolysis Affects the Techno-Functional Properties of Lesser Mealworm Protein Hydrolysates

**DOI:** 10.3390/foods9040381

**Published:** 2020-03-25

**Authors:** Giulia Leni, Lise Soetemans, Augusta Caligiani, Stefano Sforza, Leen Bastiaens

**Affiliations:** 1 Department of Food and Drug, University of Parma, 43124 Parma, Italy; giulia.leni@studenti.unipr.it (G.L.); lise.soetemans@vito.be (L.S.); augusta.caligiani@unipr.it (A.C.); stefano.sforza@unipr.it (S.S.); 2 VITO, Flemish Insititute for Technolgical Research, 2400 Mol, Belgium

**Keywords:** edible insect, protein hydrolysate, enzymatic hydrolysis, degree of hydrolysis, techno-functional properties, novel proteins

## Abstract

Protein hydrolysates from lesser mealworm (*Alphitobius diaperinus*, LM) were obtained by enzymatic hydrolysis with protease from *Bacillus licheniformis*. A preliminary test performed for five hours of hydrolysis generated an insect protein hydrolysate with 15% of degree of hydrolysis (DH), optimum solubility property and oil holding capacity, but emulsifying and foaming ability were completely impaired. In order to investigate the potential implication of DH on techno-functional properties, a set of protein hydrolysates with a different DH was obtained by sub-sampling at different time points during three hours of enzymatic hydrolysis process. An increase in DH% had positive effects on the solubility property and oil holding ability, while a reduced emulsifying ability was observed up to five hours of hydrolysis. These results demonstrated that the enzymatic hydrolysis, if performed under controlled conditions and not for a long period, represents a valid method to extract high quality protein from insects with tailored techno-functionality, in order to produce tailored ingredients for feed and food purpose.

## 1. Introduction

According to the last Food and Agriculture Organization (FAO) reports, a huge effort must be deployed to meet the future food demand connected to the increasing world population. The world in 2050 will host about 9 billion people, 30% higher than nowadays, and a strong lack of farmland, water, nutrients, and non-renewable energy is expected [1]. Along the food chain, the meat production represents the most impacting field and for these reasons novel food protein sources have been envisioned to meet the future demand [2].

Insects represent a good source of proteins for feed and food applications. In fact, the amount of proteins in insects can range between 30% and 60% on dry matter basis, with a high-quality profile rich in essential amino acids [3,4]. Furthermore, insects, in comparison to the common livestock, are characterized by many environmental advantages, such as less land use, feed, and water requirement, and a high food conversion ratio [5]. In the European Union, the legalization on insect proteins in food and feed applications has been granted by including them in the novel food category and by permitting their use for pet, fur and aquaculture [6,7,8], respectively.

As future protein source, insects have been studied and different extraction protocols for proteins were explored. For examples, Yi et al. performed a chemical extraction on *Tenebrio molitor* by combining concentrated salt and alkali pH for an overnight extraction [9]. Bußler et al. and Zhao et al. applied an alkali extraction only after defatting the insects with hexane or ethanol [10,11]. Soetemans et al. reported the use of organic acids to obtain protein and lipid enriched fractions from black soldier fly larvae, after a mechanical removal of chitin [12]. Caligiani et al. compared the ability of three different protocols to fractionate and separate the main components of insect: Chitin, lipid, and protein. The protein fraction of black soldier fly was recovered with chemicals, by following both an alkali extraction and the milder Osborne fractionation, and enzymatic processes [4]. Indeed, the enzymatic method constitutes an essential part of the processes used by modern companies to produce, from complex matrices, a large and diversified range of products for human and animal consumption [13]. The use of exogenous enzymes, instead of chemicals, allows not only to control the process, but also to prevent protein degradation due to milder reaction condition. Proteases are efficient to separate proteins from lipids and insoluble compounds (e.g., fibers) by hydrolyzing peptide bonds and releasing peptides and free amino acids in solution with high efficiency [14]. Peptides, compared to the parental proteins, are characterized by an enhanced in gastro-intestinal digestibility and bio-accessibility [15]. In literature many studies have investigated the bioactivity of protein hydrolysates obtained from insects, such as antioxidant, angiotensin-converting enzyme-inhibitory, antidiabetic, and antihypertensive activity [16]. On the other side, limited publications are available regarding the techno-functional properties of insect protein hydrolysates. Purschke et al. [17] demonstrated the ability of targeted enzymatic hydrolysis to produce protein hydrolysates from *Locusta migratoria* protein flour with tailored techno-functional properties. They observed an increase in protein solubility, emulsifying activity, foam ability, and oil binding capacity in a broad spectrum of pH. Hall et al. [18] proved that Alcalase hydrolysis could represent an efficient biotechnology tool to improve the techno-functionality of cricket proteins. In particular, they demonstrated the ability of this enzyme to enhance the solubility, emulsion and foam capacity of insect protein hydrolysates obtained at different enzyme concentrations and time of hydrolysis.

Enzymatic assisted extraction has been demonstrated to be a valid method to extract proteins from insects in form of peptides. In our previous work, we have tested on lesser mealworm (*Alphitobius diaperinus*, LM) seven different enzymes from microbial, vegetable and animal origins, by performing at a laboratory scale an end-point hydrolysis [14]. The hydrolysates were characterized for the degree of hydrolysis (DH), the yield of extraction and the presence of free amino acids, but for their future involvement as insect-based protein ingredients in food or feed formulations, it is necessary to assess also their techno-functional properties. For the first time, the present work investigates the techno-functional properties of protein hydrolysates isolated from LM, focusing on the effect of the DH.

## 2. Materials and Methods

### 2.1. Insect Samples

LM, provided by Protifarm (Ermelo, The Netherlands), were reared as described in our previous work [14]. Larvae were killed by liquid nitrogen, packed under vacuum sealed and frozen at −20 °C. After one week, samples were freeze-dried for 36 h (Christ, gamma 1−16 LSC, Osterode am Harz, Germany) and stored at −20 °C for the future analysis. Samples were grinded for 2 min with a laboratory grinder (Microtron MB 550, Kinematica, Luzern, Switzerland) at maximum speed before each analysis.

### 2.2. Enzymatic Hydrolysis for Protein Extraction

#### 2.2.1. Preliminary Enzymatic Assisted Extraction

The enzymatic assisted extraction of proteins was carried out by commercial protease from *Bacillus licheniformis* (≥2.4 U/g; EC Number 3.4.21.62) at optimal conditions for hydrolysis as suggested by the supplier (Sigma-Aldrich, St. Louis, MO, USA). More specifically, 200 g of dried ground larvae were mixed with 1 L of a buffer solution (Na_2_HPO_4_ 10 mM) and 0.25% of enzyme in a 2 L reactor, combined with the pH-STAT system (Metrohm, Varese, Italy) to control the pH during the reactions by the addition of NaOH 1 M. By knowing the amount of NaOH added, it was possible to back-calculate the degree of hydrolysis as described by Butrè et al. [19]. The hydrolysis reaction was performed for five hours and after this time the solution was heated at 90 °C for 5 min for enzyme inactivation. The hydrolysates were then centrifuged (Eppendorf, 5810/5810 R, Milano, Italy) at 2683 g at 4 °C for 30 min. The supernatant was separated from the pellet and lyophilized with LIO-5PDGT freeze-dryer (5pascal, Milano, Italy). The freeze-dried protein hydrolysate was then defatted with diethyl ether, followed by quantification for protein content with Kjeldhal analysis, as described by Leni et al. [14].

#### 2.2.2. Set of Protein Hydrolysates Collected at Different Time-Points

The enzymatic hydrolysis as previously described, was performed again in order to obtain a set of hydrolysates collected at different time-points. More specifically, 1.5 kg of dried ground larvae were mixed with 7.5 L of a buffer solution (Na_2_HPO_4_ 10 mM) in a 10 L flask at pH 7.5 and at 60 °C. Before addition of enzyme, the mixture was homogenized in an incubator (New Brunswick, Innova 42 shaker, Eppendorf, Milano, Italy) at 130 rpm for 30 min, while checking the pH (ProfiLine pH 3310, WTW, Xylem Analytics LLC, Weilheim, Germany). The pH fluctuations were adjusted by adding NaOH 50%. After this period, 1.5 L of solution was subsampled and collected as control (time 0). Next, 0.25% of enzyme was added and the hydrolysis was performed for 180 min, with sub-sampling of 1.5 L aliquots of hydrolysate at different time points, being after 30 min, 60 min, 120 min, and 180 min. Each sub-sample, control included, was heated at 90 °C for 5 min to inactive the endogenous and exogenous enzymes and subsequently centrifuged at 4 °C for 30 min at 3220 g. The supernatant was separated from the pellet and the lipid upper layer using a 500 µm sieve and were freeze-dried to generate hydrolysates for the further analysis. Each hydrolysis reaction was performed in duplicate.

#### 2.2.3. Bulk Composition

The hydrolysates and the intact larvae were characterized in terms of humidity, lipids, total N and ash. The dry matter content was determined after drying of the samples in oven at 105 °C for 24 h. Total ash was determined after mineralization at 550 °C for two times 5 h. For crude lipid quantification an automatized Soxhlet extractor (SER 148/3 VELP SCIENTIFICA, Monza e Brianza, Italy) was used with diethyl ether. The total N content was measured by Vario EL Cube (Elementar, Langenselbold, Germany) instrument by the supplier. Briefly, the sample was burned in an oxygen rich environment at 1150 °C in the combustion tube. All the burning gasses flowed through the reduction tube (helium as support gas) and were reduced to N_2_, CO_2_, H_2_O, and SO_2_. These different components were adsorbed at Selective Trap Columns and separated liberated (purge and trap technique). The detection of the components was performed with a thermo conductivity detector (TCD) cell. The proteinaceous *N* contribution was separated from the chitinous one, assuming that 87% of total *N* in LM was from protein origin, and then multiplied for the *N* to protein conversion factor 5.67, as we described in our previous work [14]. For each sub-sample, the protein content (g/100 g DM) was calculated, as well as the protein concentration as described in Equation (1):

(1) $$\mathrm{Protein}\;\mathrm{concentration}\;\left(\frac{g}{L}\right)=\frac{g\;\mathrm{of}\;\mathrm{protein}\;\mathrm{in}\;\mathrm{the}\;\mathrm{sub}-\mathrm{sample}\;\mathrm{after}\;\mathrm{freeze}\;\mathrm{dry}\;\mathrm{expressed}\;\mathrm{on}\;\mathrm{DM}\;}{L\;\mathrm{of}\;\mathrm{sub}-\mathrm{sample}\;\mathrm{collected}}$$

#### 2.2.4. Degree of Hydrolysis

The DH, defined as the percentage of cleaved peptide bonds in a protein hydrolysate, was calculated using o-phtaldialdehyde (OPA) as described by Leni et al. [14] with some modification. In particular, the supernatants were diluted in a 2% (*w*/*v*) sodium dodecyl sulphate, stirred for 20 min and stored at 4 °C overnight before the assay. The OPA/NAC (N-acetyl-cysteine) reagent (100 mL) was prepared by combining 10 mL of 50 mM OPA (in methanol) and 10 mL of NAC 50 mM, 5 mL of 20% (*w*/*v*) SDS, and 75 mL of borate buffer (0.1 M, pH 9.5). The reagent was covered with aluminum foil to protect it from light and allowed to stir for at least 1 h before use. The OPA assay was carried out (in triplicates) by the addition of 5 μL of sample (or standard) to 215 μL of OPA/NAC reagent in microplates. The absorbance of this solution was measured, after 10 min of shacking, at 340 nm with Tecan Infinite^®^ 200 PRO spectrophotometer (Tecan, Männedorf, Switzerland) against a control cell containing the reagent and 5 μL of the buffer used for the sample. The intrinsic absorbance of the samples was measured before OPA addition and subtracted. The standard curve was prepared using L-isoleucine (0−2 mg/mL). The DH was calculated as the ratio between the free nitrogen groups after hydrolysis and the total nitrogen groups: DH% = (N free/N total) × 100. The first value was calculated by the OPA reactivity. The total moles of nitrogen atoms involved in peptide bonds before hydrolysis were calculated by considering that the 87% of total N [14] in insects is from protein origin and is involved in peptide bonds.

### 2.3. Techno Functional Properties

#### 2.3.1. Solubility

The protein solubility was determined at pH 3, pH 5, and pH 7. The hydrolysates collected at different time points, were solubilized in demineralized water till a final concentration of 1% (*w*/*w*). The pH was adjusted with NaOH and HCl and the solution was mixed with an overhead shaker (Trayster digital, IKA, Königswinter, Germany) for 30 min. The pH was checked and adjusted if needed and the samples were placed on an overhead shaker for another 30 min. After that, the tubes were weighted and centrifuged at 5910 g for 20 min at 4 °C. The supernatant was separated and collected for the analysis in order to quantify the soluble N and the ionic strength. The total *N* was determined by a chemiluminescence detector (Multi N/C 3100 Analytik Jena, Jena, Germany). Briefly, the sample was oxidized by catalytic combustion in an oxygen atmosphere at 800 °C, to N oxides. The formed measuring gas was dried and entered in the reaction chamber of the chemiluminescence detector. There, the N monoxide present in the measuring gas, was oxidized with ozone into activated N dioxide. By emitting light photons (luminescence) the molecules of the N dioxide returned to their original state. The luminescence, proportional with the N monoxide concentration, was detected using a photomultiplier. The ionic strength was measured with a conductivity meter (ProfiLine Cond 3310, WTW, Weilheim, Germany).

The protein solubility was calculated as described in Equation (2):

(2) $$\mathrm{Protein}\;\mathrm{solubility}\;\left(\%\right)=\frac{g\;N\;\mathrm{in}\;\mathrm{the}\;\mathrm{supernatant}\;}{g\;\mathrm{proteinaceous}\;N\;\mathrm{in}\;\mathrm{the}\;\mathrm{sample}}\;\times \;100$$

The N-content in the supernatant measured by chemiluminescence analysis was assumed to be only from protein origin, whereas the amount of proteinaceous *N* in the sample was determined as described in 2.2.3. The analysis was performed in triplicate. In order to compare the results obtained with food standard proteins, the procedure was repeated with egg white (Sigma-Aldrich, St. Louis, MO, USA).

#### 2.3.2. Emulsification Properties

The emulsification property was determined following the method proposed by Purschke et al. [17] with some modification. Briefly, the hydrolysates were diluted with demineralized water at a concentration of 0.1% (*w*/*w*) and mixed with an overhead shaker for 30 min. The ionic strength was measured with a conductivity meter (ProfiLine Cond 3310, WTW, Weilheim, Germany). The solution was centrifuged at 3220 g for 15 min and the supernatant mixed with commercial corn oil (Vandemoortele, Ghent, Belgium) (1:1 *v*/*v*) and emulsified at 11.000 rpm for 30 s using a homogenizer (ULTRA-TURRAX^®^ T18, IKA, Königswinter, Germany). An aliquot of the emulsion was immediately transferred into scaled tubes and centrifuged at 3220 g for 15 min at 20 °C (fixed angle centrifuge). The height of the resulting emulsified layer (Hel), even if not homogeneous in the scaled tube, and the total height of solution (Hs) were used to calculate the emulsification ability as described below (3). The analyses were performed in triplicate.

(3) $$\mathrm{Emulsifying}\;\mathrm{activity}\;\left(\%\right)=\frac{\;\mathrm{Hel}}{\mathrm{Hs}}\;\times \;100$$

Casein from bovine milk and egg white (Sigma-Aldrich, St. Louis, MO, USA) were subjected to the same procedure in order to evaluate and compare their emulsification properties with insect protein hydrolysates.

#### 2.3.3. Oil Holding Capacity

For the oil holding capacity (OHC), 1 g of hydrolysate was transferred to a falcon tube and 10 g of commercial corn oil (Vandemoortele, Ghent, Belgium) were added. The solution was mixed with on overhead shaker for 5 min at 55 rpm. After 30 min, the tube was centrifuged at 3000 g for 30 min at 20 °C. The sample was re-weighed after 10 min of decantation upside-down (45° angle) and the holding capacity calculated as described in the equation below (4):

(4) $${\mathrm{OHC}}_{\left(g\;\mathrm{oil}/g\;\mathrm{sample}\right)}=\frac{W2-W1}{W0}$$

where W0 was the weight of the sample, W1 was the weight of the tube and the sample, W2 was the weight of the tube after decantation. The analyses were performed in triplicate. In order to compare insect protein hydrolysate with standard food proteins, egg white, and casein from bovine milk (Sigma-Aldrich, St. Louis, MO, USA) were subjected to the same procedure and their OHC determined.

#### 2.3.4. Foaming Capacity

The foaming capacity were measured with a homemade apparatus, composed by a graduated glass cylinder, in which was placed the solution, and a pump which fluxed air inside the mixture. The hydrolysate was suspended in demineralized water at a final concentration of 1% and mixed on an overhead shaker for 30 min. The ionic strength was measured with a conductivity meter (ProfiLine Cond 3310, WTW, Weilheim, Germany) and then the solution was transferred in the foam tube and the starting volume (in cm) was noted. The air flow was bubbled through the sample at a flow rate of 2 L/h for 1 min and the final volume reached by the foam was red after pump stopping and reported. Casein from bovine milk and egg white (Sigma-Aldrich, St. Louis, MO, USA) were subjected to the same procedure for evaluating and comparing their foaming capacity with insect protein hydrolysates. The analysis was performed in triplicate. The foam capacity was measured with the following Equation (5):

(5) $$\mathrm{Foam}\;\mathrm{capacity}\;\left(\%\right)=\frac{\mathrm{Volume}\;\mathrm{foam}}{\mathrm{Volume}\;\mathrm{start}}\;\times \;100$$

### 2.4. Statistical Analysis

Data are expressed as the mean ± standard deviation. Statistical analysis was performed using SPSS version 21.0 (SPSS Inc., Chicago, IL, USA). The data were subjected to one-way analysis of variance (ANOVA) to determine the differences between samples. Significant differences were compared at a level of *p* < 0.05.

## 3. Results

### 3.1. Techno Functional Assay of Protein Hydrolysate

Insect protein hydrolysate was obtained from LM with the protease from *Bacillus licheniformis*. The whole insect starting material contained 52 ± 0.2% of proteins on dry matter basis. After five hours of hydrolysis, a hydrolysate with a DH of 14.9 ± 0.2% and protein concentration of 51.39 ± 0.02 g/L was obtained. The final protein hydrolysate, after the defatting step, contained 58.2 ± 1.3% of proteins on dry matter basis (Table 1). The protein hydrolysate presented a high solubility property, with 95 ± 4% of total proteins soluble at pH 3, 5, and 7, explicable by the presence of peptides and free amino acids. Further, the protein hydrolysate presented also good capacity to hold oil, with 6.7 ± 0.6 g oil per g of sample. This value was 5 times more than the ability evaluated for casein and egg white (Table 2). On the contrary, the protein hydrolysate did not display foaming ability and only a slight capacity to form emulsions. In order to better explore if the high DH% reached could have affected foaming and emulsifying property, additional enzymatic hydrolysis were performed aiming at generating hydrolysates with different DHs.

### 3.2. Generation of Protein Hydrolysates with Different Degrees of Hydrolysis

Via subsampling during enzymatic assisted hydrolyses of LM starting material, water soluble protein hydrolysates with a DH ranging between 2.9% and 9.8% were obtained. The water-soluble fraction of the control sample, even if obtained without the enzyme addition, presented itself a small DH%, which could be related to a mild denaturation and hydrolysis occurred during the heat inactivation. This is in accordance with the findings of Purschke et al. [17] and Hall et al. [18], who both demonstrated an initial DH of about 5%. No significant differences were determined on DH% from 30 min to 60 min of hydrolysis, while a significant increase was monitored from 60 min till 180 min.

The proximate composition of the soluble fraction of the control and the hydrolysates was determined in terms of humidity, protein, lipid, and ash content and is reported in Table 1. All samples presented a similar compositional profile, rich in protein (66 ± 4% on DM on average) and with a slight amount of lipid (on average 15 ± 4% on DM basis).

**Figure 1:**
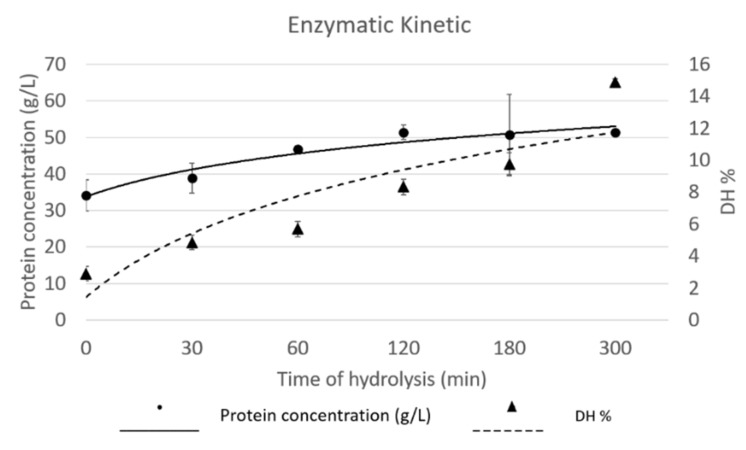
Enzymatic kinetic described by protein concentration (g/L) and DH% of hydrolysates collected at different time points.

The addition of exogenous enzyme increased the protein concentration in the supernatant of each sub-sample, even if, after 120 min, it was determined a flatting of rise near to a plateau, as reported in the enzymatic kinetic curve in Figure 1. Nevertheless, the protein hydrolysate collected after 180 min of hydrolysis had a protein concentration 48% higher than the one of control sample, thus collected before enzyme addition.

The enzymatic kinetic is also here described by evaluating the increase in DH% during the hydrolysis (Figure 1). By combining the slopes of the two curves, it is possible to assume that enzyme, during the hydrolysis, preferred to act on protein material, which was already extracted and in solution, rather than extract more protein material from the residual insect biomass. In fact, if the protein concentration did not show significative differences between the different hydrolysates, the results from DH% underlined a significative increase of free amino groups, which can be described by the hydrolysis of protein which are already in solution.

### 3.3. Functional Properties

#### 3.3.1. Solubility

The solubility property in function of pH is an important parameter of the protein hydrolysates in view of their potential industrial application. Solubility of proteins relates to surface hydrophobic (protein–protein) and hydrophilic (protein–solvent) interaction; in food case, such solvent is the water, and therefore the protein solubility is classified as a hydrophilic property [20]. In the present work, all hydrolysates were more soluble than native proteins at both pH 3 and pH 7 with values higher than 82%, even if only after 60 min of hydrolysis the difference became significant. In general, all samples, control included, presented better solubility at these pH than at pH 5 (Table 2). The lower solubility at pH 5 is due to the proximity to the isoelectric point. This is in accordance with what reported by Bußler et al. [10], who discovered that *Tenebrio molitor* intact proteins presented lowest solubility round pH 4. Purschke et al. identified at pH 5 the isoelectric point of protein hydrolysate obtained from *Locusta migratoria,* belonging to a different insect species [17].

The proximity to the isoelectric point could also justify the high standard deviation (15% on average) of trials performed at pH 5. In fact, at isoelectric pH, the zero net charge reduces the repulsive electrostatic forces, whereas the attraction forces predominate and cause the protein aggregation and precipitation. In the hydrolysate produced after 300 min of hydrolysis no significant differences were identified at pH 3, 5, and 7. This is in agreement with wat reported by Hall et al. [18] where cricket hydrolysate produced after 90 min with 1.5% of Alcalase enzyme did not present big differences at pH 3, 7, 8, and 10.

The % of solubilized protein in the current study was higher compared to Purschke et al., [17] where the solubility of *Locusta migratoria* protein hydrolysates obtained from Neutrase and Flavourzyme did not exceed the 6%. The differences could be mainly related to the different enzymes used for the hydrolysis, which are characterized by a diverse cleavage specificity. Instead, Hall et al. obtained protein hydrolysates from Alcalase activity on cricket proteins with a solubility profile similar to our results [18].

**Figure 2:**
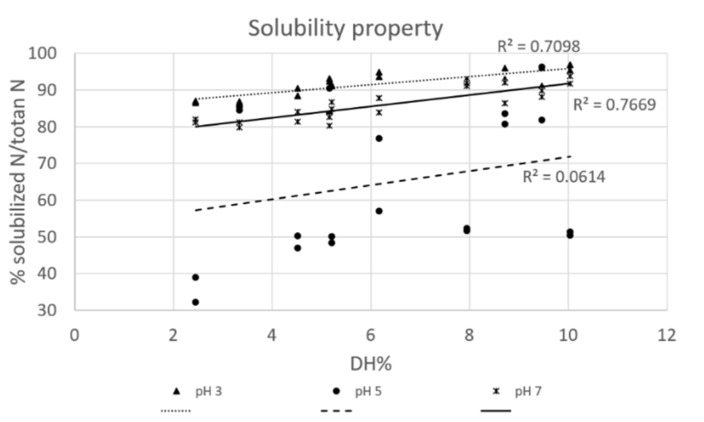
Solubility properties of 1% of protein hydrolysates (average ionic strength 2.8 ± 0.2 mS/cm) reported as % of solubilized *N* in function of DH%.

In Figure 2 the % of solubilized protein at different pH was plotted against the DH% and a positive correlation was observed for solubility at pH 3 and 7 (respectively, *r* = 0.842 and *r* = 0.876), whereas close to the isoelectric point, protein solubility stays mostly unchanged. An increase in solubility with increasing DH-values may be associated with an increase of small peptides, that exposes more ionizable amino and carboxylic groups. These groups promote electrostatic repulsion and enhances the formation of hydrogen bonds with water molecules and, as such, solubility improvement [21]. This positive correlation was also determined by Purschke et al. when Alcalase, Neutrase and Papain were used to produce protein hydrolysates from locust, while Flavourzyme did not show any correlation at pH 3, 5, 7, and 9. The latter maybe due to the different proteolytic activity of this enzyme [17]. The same correlation was also determined for other animal and vegetable matrices, such as proteins originating from salmon and egg hydrolyzed with Alcalase [22,23], rice endosperm and chickpea hydrolyzed respectively with endoprotease and Alcalase [21,22,23,24]. At pH 5 we did not determine any correlation, due to the instable peptide solubility near the isoelectric point. The same phenomenon was underlined for sardinella hydrolyzed with Alcalase, where no correlation between DH and solubility was determined near the isoelectric point determined at pH 3 and 4 [25].

#### 3.3.2. Emulsifying Activity

**Figure 3:**
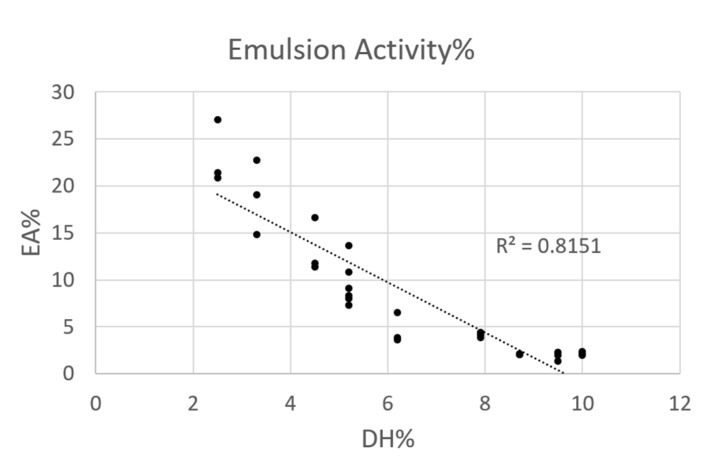
Emulsifying activity of 0.1% of protein samples (average pH 7.5 ± 0.2 and ionic strength of 1.3 ± 0.1 mS/cm) reported in function of DH%. The analysis was done in triplicate. Note: emulsification layers were incomplete—values only given as indication of a trend, not for direct comparison with literature data.

Emulsions consist of two immiscible phases, and the oil in water emulsions are the most common in food products. In the present work, insect protein hydrolysates were characterized by an incomplete emulsion layers, which, nevertheless, have been considered in order to give an indication of trends. The control sample presented the highest emulsifying ability (EA), which significantly decreased immediately after the enzyme addition. In particular, as illustrated in Figure 3, a negative correlation (*r* = 0.903) was recorded between emulsifying activity and DH% underlining the loss of this property during the hydrolysis, till manifesting the complete coalescence of oil droplets and phases separation. 

The protein emulsification mechanism is attributed to their migration to the surface of freshly formed oil droplets during homogenization. Proteins are able to form a protective film promoting oil-in-water emulsion due to their duality for the presence of hydrophilic and hydrophobic groups [26]. The shorter peptides, released from proteins after hydrolysis, may migrate to the interface oil/water more rapidly than proteins, but they are less efficient to reduce the interfacial tension between the two phases and to create a strong interfacial film round oil droplets [26]. Similar results were found by Quaglia and Orban [27], Kristinsson and Rasco [28], and Purschke et al. [17] working with sardine, salmon, and cricket, respectively. In this last case, a significant loss in emulsifying activity (mainly at acidic pH) in comparison to the control sample was observed when cricket protein flour was hydrolyzed with Neutrase and Flavourzyme. At neutral pH the emulsifying activity of protein hydrolysates obtained from the two enzyme activities displayed an opposite behavior. In fact, Neutrase negatively affected the emulsify ability, while Flavourzyme improved it, maybe due to the different specificity of proteolytic cleavage. Hall et al. [18] identified the same correlation when 0.5% of Alcalase/substrate concentrations were used, while at higher enzyme concentrations (3%) no correlation was identified.

#### 3.3.3. Oil Holding Capacity

**Figure 4:**
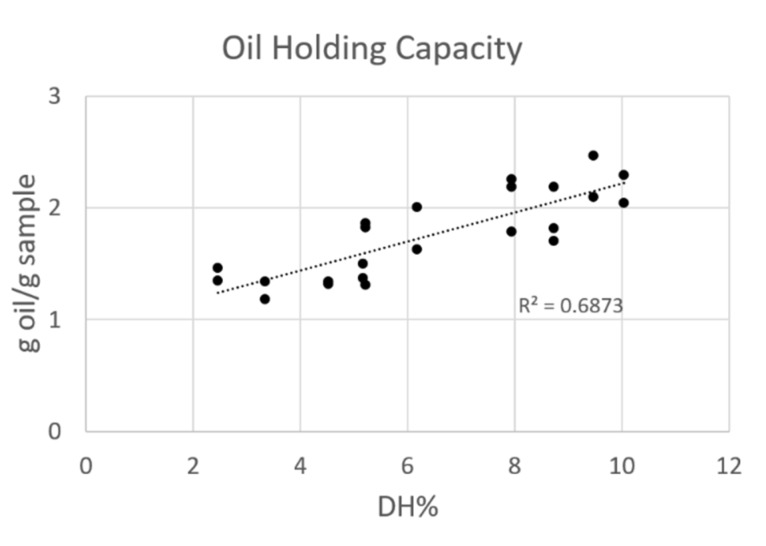
Oil holding capacity of the different hydrolysates and controls produced expressed as g oil/g sample in function of DH%. The analysis was done in triplicate.

The oil holding capacity (OHC) of control and hydrolysates was correlated to the different DH% and reported in Figure 4. The lowest value, 1.4 ± 0.1 g oil/g sample, was determined for the control sample. This amount increased with an increasing DH% till the 2.2 ± 0.2 g oil/g sample only after 180 min of reaction and has expected to continuously increase. In fact, as determined in the preliminary test, after 300 min of reaction the capacity to hold oil increased till 6.7 ± 0.6 g oil/g sample.

This raise could be explained by the modification of protein structure and the exposure of more hydrophobic side chain of amino acids, which, before the hydrolysis, were trapped in the protein folding, promoting the physical entrapment of oil [29]. Purschke et al. demonstrated the improvement in OHC of protein hydrolysates when compared to the unhydrolyzed samples even if no correlations with DH were calculated [17]. Souissi et al. obtained results in agreement with Purschke, demonstrating the increased ability of sardinella protein hydrolysate to hold oil, if compared to intact proteins [25]. Several authors observed in plant protein hydrolysates an initial increase in OHC upon hydrolysis [28]. However, a critical point exists at which the liberation of polar ionizable groups has a larger impact on OHC, than the increased availability of hydrophobic regions [30]. 

#### 3.3.4. Foaming Capacity

**Figure 5:**
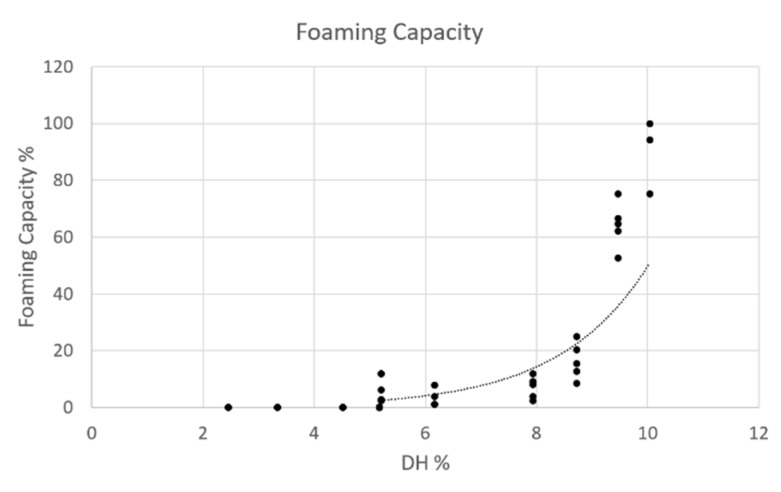
Foaming capacity of 1% of protein samples (average pH 7.9 ± 0.3 and ionic strength of 2.8 ± 0.2 mS/cm) reported in function of DH% with the trendline. The analysis was done in triplicate.

Foam is a colloidal system comprising a continuous aqueous phase with dispersed gas. Proteins, due to their amphiphilic nature, represent a good surfactant with the hydrophobic portion oriented to the air bubbles and the hydrophilic part to the watery phase [24]. In Figure 5, the foaming capacity of the different hydrolysates, with a comparable average pH and ionic strength, was plotted to their DH%, along with the control and an exponential correlation was determined. The control sample did not present foaming capacity, which on the contrary seemed to appear in the hydrolysates collected after 60 min of hydrolysis, reaching their maximum after 180 min of hydrolysis. Nevertheless after 300 min of hydrolysis no foam capacity was determined, defining this exponential trend only up to limit value of DH, which was between 10% to 15%.

An increase in DH% probably led to a more pronounced amphiphilicity, which may enhance the interfacial interaction with air bubbles until a DH of 10%. However, even if a foaming capacity was observed, all formed foams did not display any stability property and after 1 min they started to collapse. These results were in line with what reported for other protein hydrolysates obtained from edible insects [17,18].

### 3.4. Potential Application

The enzymatic hydrolysis led to an improvement of solubility and oil holding ability, while it reduced the emulsifying property. Furthermore, it was determined that functional properties could be tailored according to their DH value. Insect protein hydrolysates, compared to casein and egg white, were characterized by higher OHC. This is not of a secondary importance since the oil holding capacity is a property appreciated especially for the meat industry. In fact, the higher the OHC, the higher the ability of a food or feed formula to retain flavors and improve the palatability [31]. The high solubility property at pH 3 and 7, and the increase in oil holding capacity demonstrated the potential for using LM hydrolysates in acidic food systems, such as sports beverages and acidified sauces, and in feed system as replacement of milk for weanling animals. The enzymatic assisted extraction affected the ability of LM proteins to act as surfactants for marinating oil and air droplets dispersed in an aqueous solution. In fact, immediately after 30 min from the enzyme addition the emulsifying ability of LM protein hydrolysates started to significantly decrease. Furthermore, the emulsify property evaluated for all the insect samples were far to the ones calculated for egg white and casein, which are known to be good emulsifiers. For this reason, for feed and food emulsions, which will be prepared with insect hydrolysates, it could be necessary to add emulsifiers to stabilize the formulation. LM proteins displayed the ability to foam after 60 min of enzymatic hydrolysis, but after 300 min of hydrolysis no foaming ability was determined. The increasing in hydrolysis time, and so DH%, could have impaired the ability of peptides to arrange round air bubbles, due to the shorter length. The maximum foam ability was determined after 180 min of hydrolysis, overcoming the foam ability of casein. Nevertheless, the formed foams did not show stability and, immediately after 1 min from their constitution, they started to collapse. For this reason, insect protein hydrolysate could not be used as foaming agents but, due to the absence of foam stability, could be included in food or feed beverages.

## 4. Conclusions

In conclusion, this work provides for the first time information about the influence of DH% on techno-functional properties of protein hydrolysates produced from LM under process condition that are scalable for the industrial production. These results demonstrated that the functional properties of LM could be tailored by enzymatic assisted extraction. Nevertheless, deep investigations are needed in order to evaluate how these properties could be affected when insect protein-based ingredients will be included in food/feed complex matrices.

## Figures and Tables

**Table 1:** Bulk composition of hydrolysates and freeze-dried control samples collected at different time points during the enzymatic hydrolysis with information about DH% and protein concentration.

Hydrolysis Time	Protein% on DM	Lipid% on DM	Ash% on DM	DH%°	Protein Concentration (g/L)
**Preliminary test**					
300 min *	58.2 ± 1.3 ^a^	nd	nd	14.9 ± 0.2 ^e^	51.39 ± 0.02 ^b^
**Detailed test**					
Control (0 min)	68.8 ± 4.9 ^b^	16.3 ± 0.9 ^ab^	10.7 ± 0.1 ^a^	2.9 ± 0.5 ^a^	34.2 ± 4.3 ^a^
30 min	69.2 ± 4.5 ^bc^	10.9 ± 6.3 ^a^	9.3 ± 1.3 ^a^	4.8 ± 0.5 ^b^	38.9 ± 4.1 ^a^
60 min	65.41 ± 1.05 ^bc^	12.7 ± 2.3 ^ab^	9.6 ± 0.8 ^a^	5.7 ± 0.5 ^b^	46.72 ± 1.01 ^ab^
120 min	63.5 ± 1.7 ^cd^	16.04 ± 2.36 ^ab^	8.66 ± 1.98 ^a^	8.3 ± 0.5 ^c^	51.4 ± 1.9 ^ab^
180 min	62.1 ± 0.3 ^d^	17.3 ± 2.6 ^b^	9.1 ± 0.6 ^a^	9.8 ± 0.7 ^d^	50.78 ± 10.96 ^ab^

Results are expressed as the mean ± standard deviation (*n* = 6). Values followed by different letters within one column are significantly different (*p* < 0.05). Abbreviation: degree of hydrolysis, DH; dry matter, DM; lesser mealworm, LM; nd, not determined; ° For samples collected from 0 to 180 min DH% was calculated by OPA assay, for sample obtained after 300 min of hydrolysis DH% was calculated from pH-STAT method; * hydrolysate produced in a separated enzymatic hydrolysis and subjected to a defatting step with diethyl-ether, as described in 1.2.1.

**Table 2:** Techno-functional properties of freeze-dried samples collected at different time: protein solubility (pH 3, 5, 7), emulsification ability, oil holding and foaming capacity. Information about casein and egg white functionality are also reported.

Sample	Protein Solubility%			Emulsification Activity%	Oil Holding Capacity g oil/g Sample	Foaming Capacity%
	pH 3	pH 5	pH 7			
**Preliminary test**						
300 min *	94.8 ± 4.8 ^ab^	96.1 ± 4.2 ^b^	94.3 ± 0.6 ^d^	1.2 ± 0.3 ^a^ **	6.7 ± 0.6 ^d^	0
**Detailed test**						
Control	86.7 ± 0.3 ^a^	60.3 ± 28.6 ^a^	81.04 ± 0.95 ^a^	20.99 ± 4.06 ^e^ **	1.4 ± 0.1 ^a^	0
30 min	91.1 ± 2.1 ^ab^	67.8 ± 22.4 ^a^	82.1 ± 1.6 ^ab^	11.8 ± 3.1 ^d^ **	1.4 ± 0.1 ^a^	0
60 min	92.7 ± 1.8 ^b^	58.1 ± 12.9 ^a^	85.8 ± 1.7 ^bc^	6.7 ± 2.7 ^c^ **	1.7 ± 0.3 ^ab^	5.3 ± 4.2 ^a^
120 min	93.6 ± 1.6 ^b^	67.1 ± 17.4 ^a^	90.4 ± 2.7 ^cd^	3.1 ± 1.1 ^bc^ **	1.99 ± 0.25 ^bc^	12.59 ± 7.02 ^a^
180 min	94.9 ± 2.6 ^b^	69.9 ± 2.6 ^a^	90.9 ± 2.5 ^d^	2.03 ± 0.36 ^b^ **	2.2 ± 0.2 ^c^	73.6 ± 16.1 ^c^
**Standard protein**						
Casein	nd	nd	nd	34.3 ± 2.3 ^f^	1.52 ± 0.03 ^a^	43 ± 5 ^b^
Egg white	85.9 ± 2.6 ^a^	80.4 ± 4.3 ^a^	86.4 ± 1.5 ^bc^	52.5 ± 3.5 ^g^	1.1 ± 0.2 ^a^	123 ± 24 ^d^

Results are expressed as the mean the mean ± standard deviation (*n* = 6). Values followed by different letters within one column are significantly different (*p* < 0.05). nd: not determined. * hydrolysate produced in a separated enzymatic hydrolysis as described in 1.2.1. ** Incomplete emulsion layers were observed—value also given as indication of trends.
